# Supplementary material for: Environmentally Friendly, High-Performance Fire Retardant Made from Cellulose and Graphite
Source: Polymers (Basel). 2021 Jul 22;13(15):2400. doi: 10.3390/polym13152400 (PMC8348208; doi:10.3390/polym13152400)
Supplement: Supplementary file 1 [file polymers-13-02400-s001.zip › polymers-1289688-supplementary.pdf]

Supplementary Materials

Environmentally Friendly, High-Performance Fire Retardant Made from Cellulose and Graphite

Leandra P. Santos [1], Douglas S. da Silva [2], Thais H. Morari [1] and Fernando Galembeck [1,2,*]

[1] Galembetech Consultores e Tecnologia Ltda., Campinas 13080-661, Brazil; leandrapereiradosantos@gmail.com (L.P.S.); thmorari91@hotmail.com (T.H.M.)
[2] Department of Physical Chemistry, Institute of Chemistry, University of Campinas, Campinas 13083-970, Brazil; fernagal@unicamp.br
* Correspondence: fernagal@unicamp.br

Annex S1: Specific optical density of smoke test and result

INSTITUTO DE PESQUISAS TECNOLÓGICAS

Laboratório de Segurança ao Fogo e a Explosões – CETAC/IPT

RELATÓRIO DE ENSAIO Nº 1 117 025-203

CLIENTE: Itaúna Administração e Engenharia Ltda.
ST de Autarquias Sul, Quadra 05, Bloco K, nº 17 Asa Sul.
CEP: 70.070-937 – Brasília/DF.

INTERESSADO: Brasil Grafeno Serviços, Engenharia e Pesquisa Ltda - BRASGRAFE

NATUREZA DO TRABALHO: Determinação da densidade óptica específica de fumaça.

REFERÊNCIA: Orçamento FIPT nº 11141/19 datado de 02.09.2019.

1 INTRODUÇÃO

O método de ensaio definido na norma ASTM E662 utiliza uma câmara de densidade óptica fechada, onde é medida a fumaça gerada por materiais sólidos. A medição é feita pela atenuação de um raio de luz em razão do acúmulo da fumaça gerada na decomposição pirolítica sem chama e na combustão com chama.

Os corpos de prova medindo 76 mm x 76 mm são testados na posição vertical, expostos a um fluxo radiante de calor de 2,5 W/cm^2. São realizados ensaios com aplicação de chama piloto, descritos como "com chama", visando garantir a condição de combustão com chama e outros sem, descritos como "sem chama", visando garantir a condição de decomposição pirolítica. Os resultados são expressos em termos de densidade óptica específica (sem unidade), Ds, de acordo com a seguinte equação:

$$Ds = V / AL\,[\log_{10}(100/T) + F];$$

Onde: V é o volume da câmara fechada, A é a área exposta do corpo de prova, L é o comprimento do caminho da luz através da fumaça, T é a porcentagem de transmitância da luz e F é uma função da densidade óptica do filtro utilizado.

Os resultados do ensaio estão apresentados nas formas tabular e gráfica neste relatório. De acordo com a norma, os ensaios são conduzidos até um valor mínimo de transmitância ser atingido, agregando-se, no mínimo, um tempo adicional de ensaio de três minutos, ou até o tempo máximo de ensaio de 20 minutos, o que ocorrer primeiro.

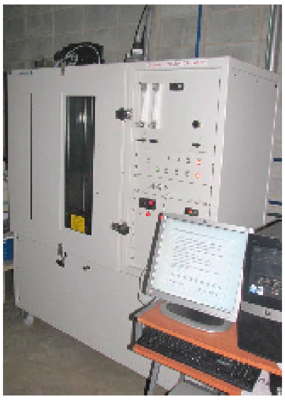

Fotografia 1 – Câmara de ensaio

Os resultados apresentados neste documento se aplicam somente ao item ensaiado ou calibrado.
Este documento não dá direito ao uso de nome ou da marca IPT, para quaisquer fins, sob pena de indenização.
A reprodução deste documento só poderá ser feita integralmente, sem nenhuma alteração.

Laboratório de Segurança ao Fogo e a Explosões – CETAC/IPT
Laboratório de Ensaio Acreditado pela Cgcre de acordo com a ABNT NBR ISO/IEC 17 025 sob o número CRL 0111

2 ITEM / MATERIAL

Foram entregues os materiais denominados "Placa de madeira maciça Pinus sem tratamento (crua)" e "Placa de madeira maciça Pinus tratada com Cellgraphe" com as seguintes características determinadas por este laboratório e descritas na tabela abaixo:

Características dos itens		
**Identificação dos itens**	279-20	282-20
**Denominação**	Placa de madeira maciça Pinus sem tratamento – crua (Fotografia 2)	Placa de madeira maciça Pinus tratada com "Cellgraphe" (Fotografia 3)
**Espessura média (mm)**	15	16
**Massa específica aparente média (kg/m³)**	477	507
**Coloração**	Marrom (natural da madeira)	Preto (do produto de tratamento)

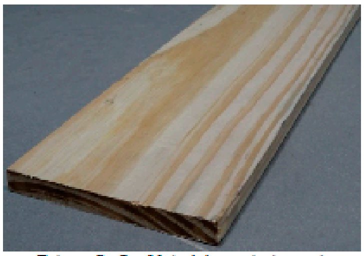
Fotografia 2 – Material sem tratamento

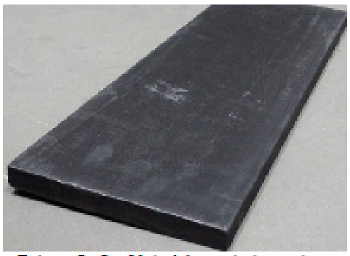
Fotografia 3 – Material com tratamento

Segundo informações do Cliente, as placas de madeira foram tratadas com o produto denominado "Cellgraphe" (código da tinta ERG-AC-13), aplicado superficialmente com espessura seca de 0,52 ± 0,04 mm.

3 MÉTODOS UTILIZADOS

- ASTM E 662: 2017a – *Specific Optical Density of Smoke Generated by Solid Materials*.

4 EQUIPAMENTOS UTILIZADOS

- Câmara de medição de densidade óptica de fumaça (identificação EQ-043).
- Balança HF-6000G (Identificação: BL-005, certificado de calibração nº 166330-101, validade: 06.2020).
- Paquímetro Digimess (PQ-006, certificado nº 169538-101, validade: 11.2021).
- Régua Arch (RG-016, certificado de calibração nº 162645-101, validade: 11.2020).

Os resultados apresentados neste documento se aplicam somente ao item ensaiado ou calibrado.
Este documento não dá direito ao uso do nome ou da marca IPT, para quaisquer fins, sob pena de indenização.
A reprodução deste documento só poderá ser feita integralmente, sem nenhuma alteração.

INSTITUTO DE PESQUISAS TECNOLÓGICAS

Laboratório de Segurança ao Fogo e a Explosões – CETAC/IPT
Laboratório de Ensaio Acreditado pela Cgcre de acordo com a ABNT NBR ISO/IEC 17 025 sob o número CRL 0111

5 RESULTADOS DE ENSAIO

Data dos ensaios: 19.03 a 20.03.2020.

5.1 Placa de madeira maciça sem tratamento – item nº 279-20

- **Densidade óptica específica (Ds) em função do tempo para queima sem chama.**

Corpo de prova	Tempo (minutos)					
	1,5	4	8	12	16	20
1	0	4	41	146	278	416*
2	0	6	60	164	306	421*
3	0	6	64	169	304	423*

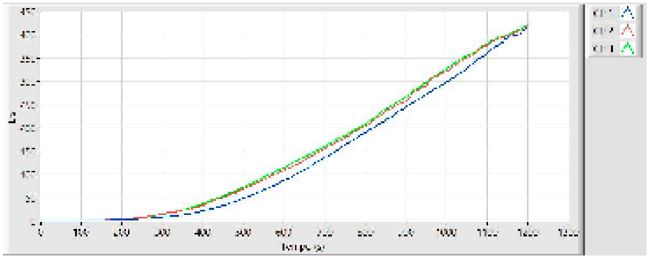

- **Densidade óptica específica (Ds) em função do tempo para queima com chama**

Corpo de prova	Tempo (minutos)						
	1,5	4	8	12	16	17,7	20
1	1	2	132	164	167	-	180*
2	0	2	19	53	112	-	209*
3	2	49	178	291	401	417*	405

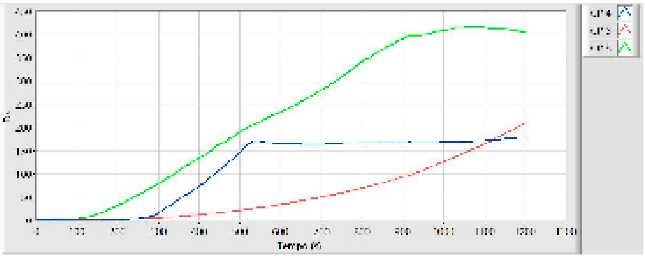

Os resultados apresentados neste documento se aplicam somente ao item ensaiado ou calibrado.
Este documento não dá direito ao uso do nome ou da marca IPT, para quaisquer fins, sob pena de indenização.
A reprodução deste documento só poderá ser feita integralmente, sem nenhuma alteração.

Laboratório de Segurança ao Fogo e a Explosões – CETAC/IPT
Laboratório de Ensaio Acreditado pela Cgcre de acordo com a ABNT NBR ISO/IEC 17 025 sob o número CRL 0111

5.2 Placa de madeira maciça tratada com "Cellgraphe"– item nº 282-20

- **Densidade óptica específica (Ds) em função do tempo para queima sem chama.**

Corpo de prova	Tempo (minutos)						
	1,5	4	8	12	16	19,5	20
1	0	9	12	50	80	-	88*
2	0	9	13	54	83	-	90*
3	0	6	10	68	82	83*	83

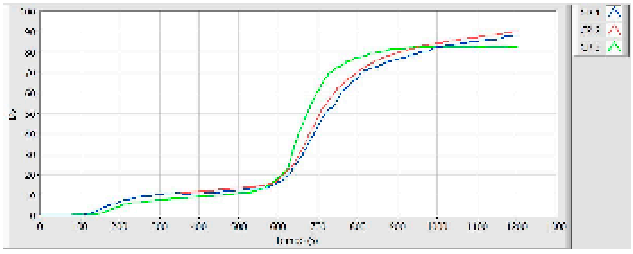

- **Densidade óptica específica (Ds) em função do tempo para queima com chama**

Corpo de prova	Tempo (minutos)					
	1,5	4	8	12	16	20
1	2	22	51	78	101	110*
2	2	24	46	61	82	100*
3	2	24	61	76	79	91*

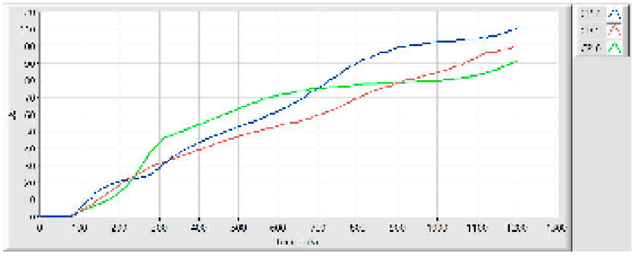

Nota 1: Os valores marcados com asterisco (*) correspondem ao índice de densidade óptica específica máxima (Dm) para cada corpo de prova.

5.3 Resultados Gerais dos Ensaios

Os valores da Tabela 1 referem-se, para cada situação de ensaio, à média de três corpos de prova (ver itens 5.1, 5.2).

Os resultados apresentados neste documento se aplicam somente ao item ensaiado ou calibrado.
Este documento não dá direito ao uso do nome ou da marca IPT, para quaisquer fins, sob pena de indenização.
A reprodução deste documento só poderá ser feita integralmente, sem nenhuma alteração.

Laboratório de Segurança ao Fogo e a Explosões – CETAC/IPT
Laboratório de Ensaio Acreditado pela Cgcre de acordo com a ABNT NBR ISO/IEC 17 025 sob o número CRL 0111

Tabela 1 – Resultados dos ensaios

Material ensaiado	Item 279-20		Item 282-20	
Tipo de Ensaio	sem chama	com chama	sem chama	com chama
**Densidade óptica específica máxima corrigida (Dm)**	**415**	**344**	**85**	**84**
Tempo, em minutos, para atingir Dm	20	19,2	19,4	20
Densidade óptica específica aos 90 s	0	1	0	2
Densidade óptica específica aos 4 min	6	21	8	24
Densidade óptica específica aos 20 min	420	346	87	101
Densidade óptica específica máxima sem correção (Ds)	421	350	87	101
Tempo, em minutos, para atingir Ds = 16	5,5	4,0	9,8	3,0
Razão máxima de desenvolvimento de fumaça (Ds/min)	69	59	32	18
Cor da fumaça	cinza	cinza	cinza	cinza

Nota 2: Os resultados relatam somente o comportamento do material ensaiado sob as condições destes métodos e os resultados não devem ser usados para indicar o risco ao fogo em outra forma ou sob outras condições. Não foi verificado se o envelhecimento do produto ou sua hidrossolubilidade comprometem o desempenho do tratamento antichama. Desta forma, há a necessidade de estabelecer a durabilidade deste acabamento para a preservação do desempenho do produto, estabelecendo, se necessário, periodicidade de sua aplicação.

6 CONCLUSÃO

O valor da densidade óptica específica máxima (Dm) atingida pela placa de madeira maciça Pinus sem tratamento (crua) foi de **415** e a placa de madeira maciça Pinus tratada com "Cellgraphe" foi de **85**, ambos correspondente ao ensaio sem chama

São Paulo, 29 de maio de 2020.

CENTRO TECNOLÓGICO DO AMBIENTE CONSTRUÍDO
Laboratório de Segurança ao Fogo e a Explosões
Eng.°Civil Mestre Carlos Roberto Metzker de Oliveira
Supervisor do Ensaio
CREA n.° 5061453656 – RE nº 08632
Assinado Digitalmente

CENTRO TECNOLÓGICO DO AMBIENTE CONSTRUÍDO
Laboratório de Segurança ao Fogo e a Explosões
Eng.º Civil Mestre Antonio Fernando Berto
Chefe do Laboratório
CREA nº 0600745569 – RE nº 2467.9
Assinado Digitalmente

Documento assinado digitalmente.
Sua validade legal e autenticidade são vinculadas às assinaturas digitais do(s) responsável(is) técnico(s) e à assinatura digital certificada do Instituto de Pesquisas Tecnológicas do Estado de São Paulo - IPT.

EQUIPE TÉCNICA

Engenheiro Civil Antonio Fernando Berto – IPT
Engenheiro Civil Carlos Roberto Metzker de Oliveira – IPT
Engenheiro Civil Anderson Nobre da Silva – FIPT
Técnico Rafael Maier da Silva – FIPT

Os resultados apresentados neste documento se aplicam somente ao item ensaiado ou calibrado.
Este documento não dá direito ao uso do nome ou da marca IPT, para quaisquer fins, sob pena de indenização.
A reprodução deste documento só poderá ser feita integralmente, sem nenhuma alteração.

Annex S2: Surface flame propagation index test and result

INSTITUTO DE PESQUISAS TECNOLÓGICAS

Laboratório de Segurança ao Fogo e a Explosões – CETAC/IPT

RELATÓRIO DE ENSAIO Nº 1 117 026-203

CLIENTE: Itaúna Administração e Engenharia Ltda.
ST de Autarquias Sul, Quadra 05, Bloco K, nº 17 Asa Sul.
CEP: 70.070-937 – Brasília/DF.

INTERESSADO: Brasil Grafeno Serviços, Engenharia e Pesquisa Ltda - BRASGRAFE

NATUREZA DO TRABALHO: Determinação do índice de propagação superficial de chama.

REFERÊNCIA: Orçamento FIPT nº 11141/19 datado de 02.09.2019.

1 INTRODUÇÃO

O método de ensaio descrito na norma NBR 9442 é utilizado para determinar o índice de propagação de chama de materiais pelo método do painel radiante, utilizando-se do equipamento visualizado na Fotografia 1.

Os corpos de prova, com dimensões de 150 ± 5 mm de largura e 460 ± 5 mm de comprimento, são inseridos em um suporte metálico e colocados em frente a um painel radiante poroso, com 300 mm de largura e 460 mm de comprimento, alimentado por gás propano e ar. O conjunto (suporte e corpo de prova) é posicionado em frente ao painel radiante com uma inclinação de 60º, de modo a expor o corpo de prova a um fluxo radiante padronizado. Uma chama piloto é aplicada na extremidade superior do corpo de prova.

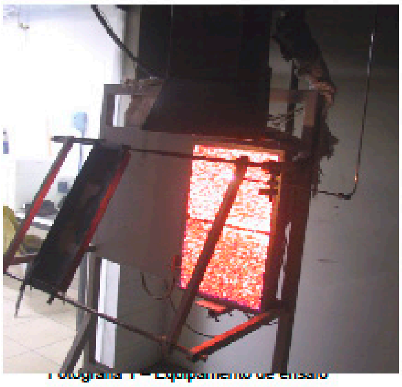

Fotografia 1 – Equipamento de ensaio

É obtido no ensaio o fator propagação de chama desenvolvida na superfície do material (Pc), medido através do tempo para atingir as distâncias padronizadas no suporte metálico com o corpo de prova, e o fator de evolução de calor desenvolvido pelo material (Q), medido através de sensores de temperatura (termopares) localizados em uma chaminé sobre o painel e o suporte com o corpo de prova.

O índice é determinado através da seguinte equação (sem unidade):

$$Ip = Pc \times Q$$

Onde:
Ip: Índice de propagação superficial de chama
Pc: Fator de propagação da chama
Q: Fator de evolução do calor.

Os resultados apresentados neste documento se aplicam somente ao item ensaiado ou calibrado.
Este documento não dá direito ao uso do nome ou da marca IPT, para quaisquer fins, sob pena de indenização.
A reprodução deste documento só poderá ser feita integralmente, sem nenhuma alteração.

Av. prof. Almeida Prado, 532 | Butantã
São Paulo | SP | 05508-901
Tel 11 3767 4000 Fax 11 3767 4002 | ipt@ipt.br
www.ipt.br

Laboratório de Segurança ao Fogo e a Explosões – CETAC/IPT
Laboratório de Ensaio Acreditado pela Cgcre de acordo com a ABNT NBR ISO/IEC 17 025 sob o número CRL 0111

2 ITEM / MATERIAL

Foram entregues os materiais denominados "Placa de madeira maciça Pinus sem tratamento (crua)" e "Placa de madeira maciça Pinus tratada com Cellgraphe" com as seguintes características determinadas por este laboratório e descritas na tabela abaixo:

Características dos itens		
Identificação dos itens	280-20	283-20
Denominação	Placa de madeira maciça Pinus sem tratamento – crua (Fotografia 2)	Placa de madeira maciça Pinus tratada com "Cellgraphe" (Fotografia 3)
Espessura média (mm)	15	16
Massa específica aparente média (kg/m³)	477	507
Coloração	Marrom (natural da madeira)	Preto (do produto de tratamento)

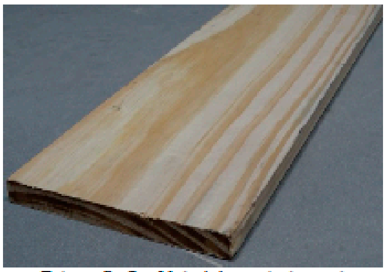
Fotografia 2 – Material sem tratamento

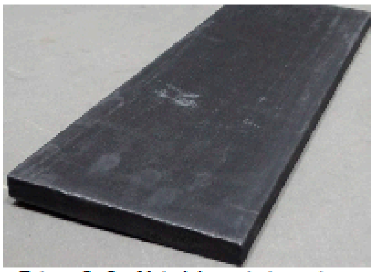
Fotografia 3 – Material com tratamento

Segundo informações do Cliente, as placas de madeira foram tratadas com o produto denominado "Cellgraphe" (código da tinta ERG-AC-13), aplicado superficialmente com espessura seca de 0,52 ± 0,04 mm.

3 MÉTODO UTILIZADO

- ABNT NBR 9442: 2019 – "Materiais de construção – Determinação do índice de propagação superficial de chama pelo método do painel radiante – Método de ensaio".

4 EQUIPAMENTOS UTILIZADOS

- Equipamento de propagação superficial de chama marca FTT (identificação: EQ-033).

Os resultados apresentados neste documento se aplicam somente ao item ensaiado ou calibrado.
Este documento não dá direito ao uso do nome ou da marca IPT, para quaisquer fins, sob pena de indenização.
A reprodução deste documento só poderá ser feita integralmente, sem nenhuma alteração.

- Balança HF-6000G (identificação: BL-005, certificado de calibração nº 166330-101, validade: 06.2020).
- Régua Hope (RG-008, certificado de calibração nº 165050-101, validade: 04.2021).
- Paquímetro Digimess (identificação: PQ-006, certificado de calibração nº 169538-101, validade: 11.2021).

5 RESULTADOS DE ENSAIO

Ensaios realizados em 27.05.2020.

Condicionamento: Os corpos de prova foram mantidos em estufa com ventilação forçada a (60 ±3)°C por 24 horas e em seguida condicionados até o equilíbrio em câmara climatizada à temperatura de (23 ± 3)°C e umidade relativa de (50 ± 5)%.

5.1 Placa de madeira maciça sem tratamento – item nº 280-20

Tabela 1 – Resultados do ensaio

Fatores	Valores		
	Médio	Mínimo	Máximo
Evolução de calor (Q)	16,6	16,6	16,7
Propagação de chama (Pc)	27,3	26,2	28,5
**Índice de propagação de chama (Ip)**	**455**	**433**	**475**

5.1.1 Observações de ensaio

- A propagação de chama avançou por toda à superfície dos corpos de prova.
- Não ocorreu gotejamento e/ou desprendimento de partículas em chama
- Desenvolvimento de fumaça de coloração cinza.

5.2 Placa de madeira maciça tratada com "Cellgraphe"– item nº 283-20

Tabela 2 – Resultados do ensaio

Fatores	Valores		
	Médio	Mínimo	Máximo
Evolução de calor (Q)	8,6	8,2	9,0
Propagação de chama (Pc)	1,9	1,4	2,4
**Índice de propagação de chama (Ip)**	**15**	**11**	**21**

Os resultados apresentados neste documento se aplicam somente ao item ensaiado ou calibrado.
Este documento não dá direito ao uso do nome ou da marca IPT, para quaisquer fins, sob pena de indenização.
A reprodução deste documento só poderá ser feita integralmente, sem nenhuma alteração.

Laboratório de Segurança ao Fogo e a Explosões – CETAC/IPT
Laboratório de Ensaio Acreditado pela Cgcre de acordo com a ABNT NBR ISO/IEC 17 025 sob o número CRL 0111

5.2.1 Observações de ensaio

- A propagação de chama avançou, em média, 160 mm (35% da superfície dos corpos de prova).
- Não ocorreu gotejamento e/ou desprendimento de partículas em chama
- Desenvolvimento de fumaça de coloração cinza.

Notas 1:

- *O índice de propagação de chama médio (Ip) foi arredondado para o múltiplo mais próximo de cinco, conforme procedimento do item 10.2 da norma de referência.*
- *Os resultados relatam somente o comportamento do material ensaiado sob as condições destes métodos e os resultados não devem ser usados para indicar o risco ao fogo em outra forma ou sob outras condições.*
- *Não foi verificado se o envelhecimento do produto ou sua hidrossolubilidade comprometem o desempenho do tratamento antichama. Desta forma, há a necessidade de estabelecer a durabilidade deste acabamento para a preservação do desempenho do produto, estabelecendo, se necessário, periodicidade de sua aplicação.*

7 CONCLUSÃO

O Índice de Propagação Superficial de Chama Médio (Ip) alcançado pela placa de madeira maciça Pinus sem tratamento (crua) foi de **455** e pela placa de madeira maciça Pinus tratada "Cellgraphe" foi de **15**.

São Paulo, 29 de maio de 2020.

CENTRO TECNOLÓGICO DO AMBIENTE CONSTRUÍDO
Laboratório de Segurança ao Fogo e a Explosões
Eng.º Civil Mestre Carlos Roberto Metzker de Oliveira
Supervisor do Ensaio
CREA n.º 5061453656 – RE nº 08632
Assinado Digitalmente

CENTRO TECNOLÓGICO DO AMBIENTE CONSTRUÍDO
Laboratório de Segurança ao Fogo e a Explosões
Eng.º Civil Mestre Antonio Fernando Berto
Chefe do Laboratório
CREA nº 0600745569 – RE nº 2467.9
Assinado Digitalmente

INSTITUTO DE PESQUISAS TECNOLÓGICAS
Documento assinado digitalmente.
Sua validade legal e autenticidade são vinculadas às assinaturas digitais do(s) responsável(is) técnico(s) e à assinatura digital certificada do Instituto de Pesquisas Tecnológicas do Estado de São Paulo – IPT.

EQUIPE TÉCNICA
Engenheiro Civil Antonio Fernando Berto – IPT
Engenheiro Civil Carlos Roberto Metzker de Oliveira – IPT
Engenheiro Civil Anderson Nobre da Silva – FIPT
Técnico André Luiz de Souza – IPT

Os resultados apresentados neste documento se aplicam somente ao item ensaiado ou calibrado.
Este documento não dá direito ao uso do nome ou da marca IPT, para quaisquer fins, sob pena de indenização.
A reprodução deste documento só poderá ser feita integralmente, sem nenhuma alteração.

Annex S3: Material classification

INSTITUTO DE PESQUISAS TECNOLÓGICAS

Laboratório de Segurança ao Fogo e a Explosões – CETAC/IPT

RELATÓRIO DE ENSAIO Nº 1 117 027-203

CLIENTE: Itaúna Administração e Engenharia Ltda.
ST de Autarquias Sul, Quadra 05, Bloco K, nº 17 Asa Sul.
CEP: 70.070-937 – Brasília/DF.

INTERESSADO: Brasil Grafeno Serviços, Engenharia e Pesquisa Ltda - BRASGRAFE

NATUREZA DO TRABALHO: Classificação dos materiais de acabamento e revestimento empregados nas edificações

REFERÊNCIA: Orçamento FIPT nº 11141/19 datado de 02.09.2019.

1 ITEM / MATERIAL

Foram entregues os materiais denominados "Placa de madeira maciça Pinus sem tratamento (crua)" e "Placa de madeira maciça Pinus tratada com Cellgraphe" com as seguintes características determinadas por este laboratório e descritas na tabela abaixo:

Características dos itens		
**Denominação**	Placa de madeira maciça Pinus sem tratamento – crua (Fotografia 1)	Placa de madeira maciça Pinus tratada com "Cellgraphe" (Fotografia 2)
**Espessura média (mm)**	15	16
**Massa específica aparente média (kg/m³)**	477	507
**Coloração**	Marrom (natural da madeira)	Preto (do produto de tratamento)

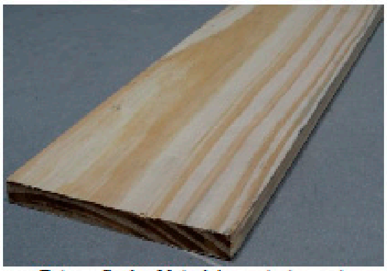
Fotografia 1 – Material sem tratamento

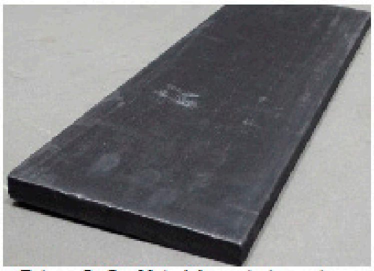
Fotografia 2 – Material com tratamento

Segundo informações do Cliente, as placas de madeira foram tratadas com o produto denominado "Cellgraphe" (código da tinta ERG-AC-13), aplicado superficialmente com espessura seca de 0,52 ± 0,04 mm.

Os resultados apresentados neste documento se aplicam somente ao item ensaiado ou calibrado.
Este documento não dá direito ao uso de nome ou da marca IPT, para quaisquer fins, sob pena de indenização.
A reprodução deste documento só poderá ser feita integralmente, sem nenhuma alteração.

Laboratório de Segurança ao Fogo e a Explosões – CETAC/IPT

2 MÉTODO UTILIZADO

- Instrução Técnica nº 10/2019 – Controle de materiais de acabamento e de revestimento. Corpo de Bombeiros da Polícia Militar do Estado de São Paulo.
- ABNT NBR 16626: 2017 – "Classificação da reação ao fogo de produtos de construção".
- Procedimento de Ensaio CETAC-LSFEx-PE 188 – Classificação dos materiais de acabamento e revestimento empregados nas edificações.

3 RESULTADOS DE ENSAIO

A Tabela 1 contem os resultados obtidos nos ensaios de reação ao fogo.

Tabela 1 – Resultados obtidos nos ensaios

**Referência**	**Índice de Propagação superficial de chama (Ip)**	**Densidade específica óptica de fumaça (Dm)**	**Gotejamento em chama (s)**
Relatório de ensaio IPT nº 1 117 025-203 referente aos ensaios nas placas de madeira maciça Pinus sem tratamento	455	415	não ocorreu
Relatório de ensaio IPT nº 1 117 026-203 referente aos ensaios nas placas de madeira maciça Pinus tratadas com "Cellgraphe"	15	85	não ocorreu

4 LIMITES ESPECIFICADOS EM NORMA

A Tabela 2 indica a classificação do material em função dos resultados nos ensaios, conforme estabelecido na Instrução Técnica nº 10 do Decreto nº 63.911 e na norma ABNT NBR 16626.

Tabela 2 – Classificação dos materiais exceto revestimento de piso

**Classes**		**ISO 1182**	**NBR 9442**	**ASTM E662**
I		Incombustível	-	-
II	A	Combustível	Ip ≤ 25	Dm ≤ 450
	B	Combustível	Ip ≤ 25	Dm > 450
III	A	Combustível	25 < Ip ≤ 75	Dm ≤ 450
	B	Combustível	25 < Ip ≤ 75	Dm > 450
IV	A	Combustível	75 < Ip ≤ 150	Dm ≤ 450
	B	Combustível	75 < Ip ≤ 150	Dm > 450
V	A	Combustível	150 < Ip ≤ 400	Dm ≤ 450
	B	Combustível	150 < Ip ≤ 400	Dm > 450
VI		Combustível	Ip > 400	-

Observações relativas à tabela 2:
Tabela retirada da Instrução Técnica nº 10 do Decreto nº 56.819 do Corpo de Bombeiros de São Paulo
Ip – Índice de propagação superficial de chama.
Dm – Densidade específica óptica máxima de fumaça

Os resultados apresentados neste documento se aplicam somente ao item ensaiado ou calibrado.
Este documento não dá direito ao uso de nome ou da marca IPT, para quaisquer fins, sob pena de indenização.
A reprodução deste documento só poderá ser feita integralmente, sem nenhuma alteração.

Laboratório de Segurança ao Fogo e a Explosões – CETAC/IPT

4.1 Classificação adicional de gotejamento em chama

Adicionalmente, a norma ABNT NBR 16626 estabelece três classificações a respeito da produção de gotejamento e/ou desprendimento de partículas em chama quando ensaiados conforme norma ABNT NBR 9442, a saber:

- d_0 se não ocorrerem gotejamento em chama e nem o desprendimento de partículas em chama;
- d_1 se não ocorrerem gotejamento em chama e nem o desprendimento de partículas em chama com duração superior a 10 s;
- d_2 se as condições anteriores não forem atendidas.

Notas 1:

- *Os resultados relatam somente o comportamento do material ensaiado sob as condições destes métodos e os resultados não devem ser usados para indicar o risco ao fogo em outra forma ou sob outras condições.*
- *Não foi verificado se o envelhecimento do produto ou sua hidrossolubilidade comprometem o desempenho do tratamento antichama. Desta forma, há a necessidade de estabelecer a durabilidade deste acabamento para a preservação do desempenho do produto, estabelecendo, se necessário, periodicidade de sua aplicação.*

5 CONCLUSÃO

O material denominado "Placa de Madeira Maciça Pinus sem tratamento (Crua)" classifica-se como **VI** de acordo com a Instrução Técnica nº 10 do Decreto Estadual de São Paulo nº 63.911 e como **VI-d_0** conforme a norma ABNT NBR 16626.

O material denominado "Placa de Madeira Maciça Pinus tratada com Cellgraphe" classifica-se como **II-A** de acordo com a Instrução Técnica nº 10 do Decreto Estadual de São Paulo nº 63.911 e como **II-A-d_0** conforme a norma ABNT NBR 16626.

São Paulo, 29 de maio de 2020.

CENTRO TECNOLÓGICO DO AMBIENTE CONSTRUÍDO
Laboratório de Segurança ao Fogo e a Explosões
Eng.º Civil Mestre Carlos Roberto Metzker de Oliveira
Supervisor do Ensaio
CREA n.º 5061453656 – RE nº 08632
Assinado Digitalmente

CENTRO TECNOLÓGICO DO AMBIENTE CONSTRUÍDO
Laboratório de Segurança ao Fogo e a Explosões
Eng.º Civil Mestre Antonio Fernando Berto
Chefe do Laboratório
CREA nº 0600745569 – RE nº 2467.9
Assinado Digitalmente

INSTITUTO DE PESQUISAS TECNOLÓGICAS

Documento assinado digitalmente.
Sua validade legal e autenticidade são vinculadas às assinaturas digitais do(s) responsável(is) técnico(s) e à assinatura digital certificada do Instituto de Pesquisas Tecnológicas do Estado de São Paulo - IPT.

EQUIPE TÉCNICA

Engenheiro Civil Antonio Fernando Berto – IPT
Engenheiro Civil Carlos Roberto Metzker de Oliveira – IPT
Engenheiro Civil Anderson Nobre da Silva – FIPT

Os resultados apresentados neste documento se aplicam somente ao item ensaiado ou calibrado.
Este documento não dá direito ao uso de nome ou da marca IPT, para quaisquer fins, sob pena de indenização.
A reprodução deste documento só poderá ser feita integralmente, sem nenhuma alteração.
